# Precursor and mature NGF live tracking: one *versus* many at a time in the axons

**DOI:** 10.1038/srep20272

**Published:** 2016-02-01

**Authors:** Teresa De Nadai, Laura Marchetti, Carmine Di Rienzo, Mariantonietta Calvello, Giovanni Signore, Pierluigi Di Matteo, Francesco Gobbo, Sabrina Turturro, Sandro Meucci, Alessandro Viegi, Fabio Beltram, Stefano Luin, Antonino Cattaneo

**Affiliations:** 1 BioSNS Laboratory, Scuola Normale Superiore and Istituto di Neuroscienze – CNR, Pisa, Italy; 2 NEST, Scuola Normale Superiore and Istituto Nanoscienze–CNR, Pisa, Italy; 3 IIT@NEST, Center for Nanotechnology Innovation, Pisa, Italy; 4 EBRI, European Brain Research Institute, Rome, Italy

## Abstract

The classical view of nerve growth factor (NGF) action in the nervous system is linked to its retrograde axonal transport. However, almost nothing is known on the trafficking properties of its unprocessed precursor proNGF, characterized by different and generally opposite biological functions with respect to its mature counterpart. Here we developed a strategy to fluorolabel both purified precursor and mature neurotrophins (NTs) with a controlled stoichiometry and insertion site. Using a single particle tracking approach, we characterized the axonal transport of proNGF *versus* mature NGF in living dorsal root ganglion neurons grown in compartmentalized microfluidic devices. We demonstrate that proNGF is retrogradely transported as NGF, but with a lower flux and a different distribution of numbers of neurotrophins per vesicle. Moreover, exploiting a dual-color labelling technique, we analysed the transport of both NT forms when simultaneously administered to the axon tips.

The reciprocal levels of Nerve Growth Factor (NGF) and of its unprocessed precursor (proNGF) play a crucial role in regulating the survival/death balance of several neuronal populations in physiopathological conditions^1^,^2^,^3^,^4^. proNGF, which is the most abundant form of NGF in the brain^5^, can either act as an intracellular or secreted precursor for mature NGF or remain unprocessed, activating survival/differentiation or apoptosis pathways, respectively. This molecular switch relies on the different receptor binding profiles, determining different biological outcomes. When administered separately or together, NGF and proNGF activate distinct and peculiar gene expression patterns in target cells^6^,^7^. Disruption of the NGF to proNGF balance has been causally linked to neurodegeneration^8^,^9^, and the proNGF *versus* NGF ratio is increased in the cortex of Alzheimer’s disease patients^5^. Thus, describing the signaling mechanisms that link NGF and proNGF cellular trafficking to their specific biological function is of crucial importance.

Axonal transport of neurotrophins (NTs) represents a crucial link between receptor mediated signaling and their biological outcome^10^. Despite its importance, we currently lack the molecular definition and characterization of NTs axonal signalling endosomes^11^. Furthermore, the question of whether and how pro-NTs move retrogradely or anterogradely along axons, in comparison to their mature counterparts, has remained so far largely unexplored. We have addressed these issues by using a biophysical approach of NT labeling and tracking in living neurons. Ideally, a quantitative comparison between the axonal transport of NGF and proNGF requires the two molecules to be fluoro-labeled at the same molecular site and with the same stoichiometry. So far, the mature forms of NTs have been chemically coupled to organic fluorophores^12^,^13^,^14^,^15^,^16^ or to biotin^17^,^18^,^19^,^20^,^21^. While this has allowed obtaining valuable information about NGF trafficking, it has not been possible to control the exact number and site of conjugated probes, so that mixed and not fully reproducible labeled protein populations are obtained. Similar approaches are not recommended for the purpose of labeling proNGF, whose pro-domain has features of an intrinsically unfolded protein^22^,^23^, nor for a detailed side-by-side comparison of the mature and precursor forms.

Here a novel fluorolabeling strategy is described, allowing for the production of “homologous” fluorescent human NGF and proNGF, based on the insertion of an 11 amino acid tag at the at the C- terminus of the protomer sequence. The inserted tag is a target for a site-specific enzymatic covalent binding and it is used here to bind a small organic dye, so that a site-specific fluorophore conjugation with 1:1 (label:NT-monomer) stoichiometry is obtained for both the unprocessed and mature forms of NGF. The technique allows for a high (≈80%) fluorescent NT production yield and for an optimal purification from the unlabeled counterparts. The obtained labelled species retain the same functional features of the native proteins. Fluorescence microscopy experiments were performed on compartmentalized living cultures of rat dorsal root ganglion (DRG) neurons, in which fluorescent proNGF and NGF were administered either separately or together. The first direct evidence that proNGF is retrogradely transported like mature NGF is provided here, although important differences of the axonal transport of the two molecules have been uncovered. Crucially, the controlled stoichiometry of the labelling reaction allowed quantifying the number of NTs in each vesicle, showing a significant difference between their distributions in the two cases. Moreover, by coadministering both neurotrophins labelled with different fluorophores, we were able to analyse the cotransport of precursor and mature neurotrophins in neurons.

## Results

### Fluo-proNGF and Fluo-NGF synthesis and functional validation

**Figure 1:**
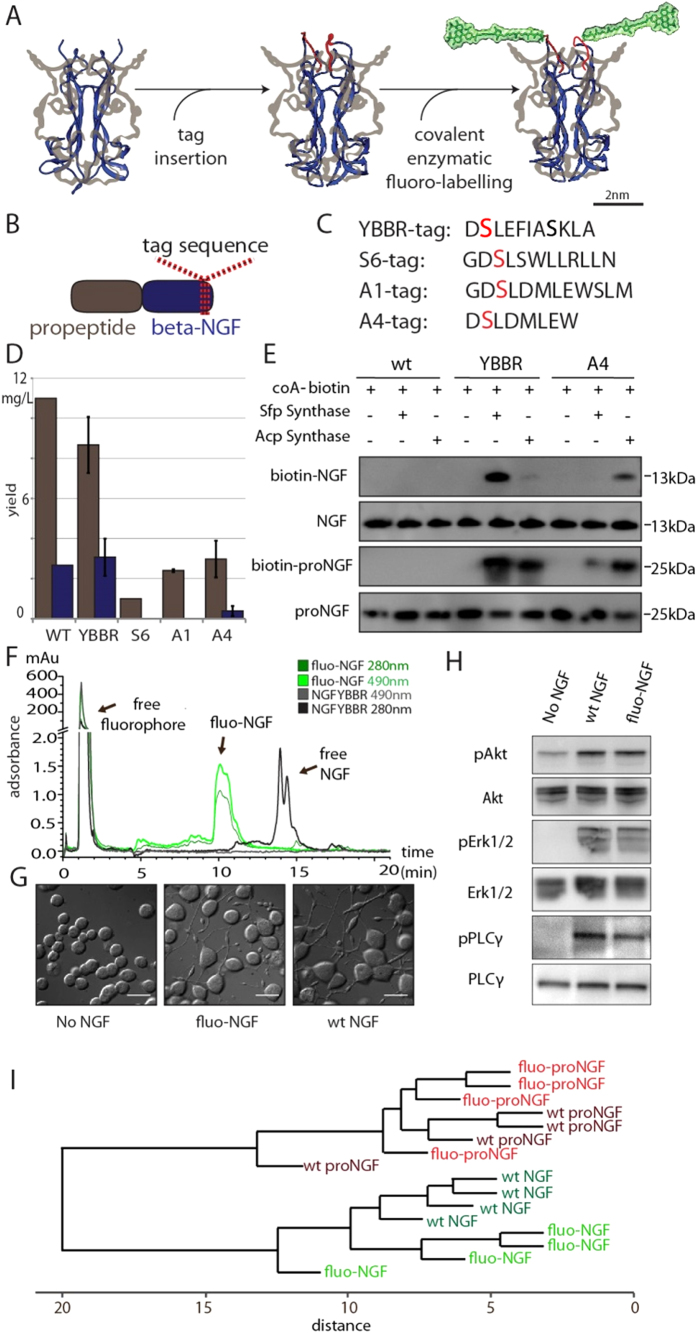
Schematic overview of production of fluorescent NTs and characterization of fluo-NGF biological activity. (**A**) Cartoon depicting the two-steps labelling strategy. Structure of human NGF (blue ribbon, PDB 1SG1) with overlaid, in grey, the pro-peptide domain^22^ (*Left)*; the tag sequence inserted at the C-terminal position of proNGF is depicted in red (*Middle*); the complete structural formula of Alexa488-maleimide-phosphopantetheinyl is added, highlighted in green (*Right*). (**B**) Scheme of proNGF sequence with highlighted tag insertion site. (**C**) Amino acidic sequence of the four screened tags, with the serine residue covalently conjugated to the fluorophore highlighted in red. (**D**) Purification yields (mg of product per litre of bacterial culture) of tagged proNGF-tag (gray) and NGF-tag (blue), compared to wt (pro)NGF. (**E**) Western blot analysis of the biotinylation reaction of purified NGF-YBBR and NGF-A4 using CoA-biotin substrate and AcpS or SfpS PPTases. The same biotinylation reaction is performed in parallel using untagged wt-NGF as negative control. Streptavidin-HRP is used for biotin detection. The anti-NGF blot (NGF and proNGF lines) is the loading control. (**F**) The HPLC chromatogram of NGF-YBBR, incubated with CoA-Alexa488 substrate in the presence and absence of Sfp-synthase, showing the different retention times of fluorescent and non-fluorescent NTs. Absorbance curves at 280 and 490 nm are reported. (**G**) Typical DIC images of PC12 differentiation assay using equimolar amounts of wt-NGF and fluo-NGF. Untreated cells are the control. Scale bars represent 20 μm. (**H**) Western blot analysis of phosphorylated Akt (pAkt), phosphorylated Erk1/2 (pErk1/2) and phosphorylated PLCγ (pPLCγ) protein levels in PC12 cells in response to wt NGF and fluo-NGF, compared to the same obtained for untreated cells (No NGF); the signal of the total corresponding signalling effectors is the loading control. (**I**) Hierarchical clustering tree of samples, corresponding to the different experimental points (for each neurotrophin type four individual PC12 cells administrations were performed). The trees show the gene expression similarity between samples. The x-axis indicates the distance between samples. Euclidean distance is the chosen metric, with average linkage clustering, using all normalized Log2 data.

We recently explored the possibility of chemically modifying NTs by the insertion of short tags derived from the acyl and peptidyl carrier proteins^24^. Following this approach, a single fluorophore per monomer of proNGF or NGF was conjugated by introducing a short amino acidic tag at the C-terminus of the proNGF sequence (Fig. 1A,B), by exploiting a site-specific enzymatic reaction to covalently link the small organic dye to a serine residue of the tag (Fig. 1C). The C-terminal portion of NTs was chosen as a permissive site to insert the tag with minimal interference with NGF structure and receptor binding^24^.

In order to optimize both expression and labelling yields, as well as to assure the maintenance of NT physiological activity, four different tag sequences were compared (spanning from 8 to 12 amino acids): YBBR, A4, A1, and S6 (Fig. 1C)^25^,^26^,^27^. The production yields obtained for all the tagged NTs after their expression, refolding and purification from *E.Coli* inclusion bodies^22^,^28^ (Supplementary Figure 1A) were compared to those obtained for the untagged counterparts (Fig. 1D): while for all tagged constructs the precursor NT could be successfully purified (although with different yields: YBBR > A4 > A1 > S6), only two of the tags (YBBR > A4) consistently yielded measurable amounts of purified mature protein. Accordingly, NGF-YBBR and NGF-A4 were further characterized. Tag insertion does not interfere with NGF functionality, according to a number of signaling, cell differentiation and proliferation assays (Supplementary Figs 1B–D). For labelling purposes, YBBR- and A4- tagged precursor or mature NGF proteins were incubated with Coenzyme A (CoA) labelled biotin substrate and Acp- or Sfp-synthase PPTases (or no enzyme as control) (Fig. 1E). Data demonstrate that a specific biotin labelling is achieved for NGF-YBBR in the presence of both enzymes, with a higher labelling yield provided by the reaction with Sfp-synthase, while NGF-A4 displays significant labelling only in presence of Acp-synthase but with a lower yield compared to the NGF-YBBR/Sfp-synthase reaction. Based on these data, YBBR sequence was identified to be the best tag for NGF and proNGF labelling.

Next, YBBR tag was exploited to produce fluorescent proNGF and NGF, hereafter referred as fluo-proNGF and fluo-NGF. To this purpose, purified proNGF-YBBR or NGF-YBBR were incubated with CoA-Alexa488 substrate in the presence of Sfp-synthase (see *Materials and Methods*). Upon labelling, fluo-proNGF and fluo-NGF were purified by ion exchange chromatography to remove both the free fluorophore and the non-reacted NT, so as to recover exclusively fluorescent NTs (Fig. 1F). The comparison of the various peaks integrals in the chromatogram allowed us to estimate a labelling yield of about 80% (see *Materials and Methods*). In order to validate whether the fluorescent NTs retain functional activity, we performed a DNA microarray analysis of gene expression activated by 1 hour treatment of PC12 cells with fluo-proNGF or fluo-NGF respectively, and compared the expression profile with those obtained after incubation of equal amounts of the corresponding *wt*, untagged counterparts. Indeed, we have previously demonstrated that this assay is able to finely discriminate proNGF from NGF functional activity in relatively short timescales, so as to minimize the impact of proNGF cleavage to NGF^6^. Clustering analysis of gene expression profiles showed that treatments with *wt* and fluorescent proNGF cluster together, while the gene expression profile obtained with *wt* NGF is farther away in the tree and is clustered together with fluo-NGF (Fig. 1I). Thus, each fluo-NGF and fluo-proNGF neurotrophin induces a global signaling very similar to that of the corresponding unmodified neurotrophin. Furthermore, fluo-NGF is able to promote PC12 cells differentiation, inducing neurite outgrowth to the same extent as wt NGF (Fig. 1G). Moreover, fluo-NGF induces a robust activation of the downstream signaling effectors involved in NGF-signaling pathways, like phosphorylated Erk1/2, PLC-γ and AKT proteins (Fig. 1H). These data indicate that fluorescent NTs are biologically active and induce physiological responses comparable to those of the respective native proteins.

### fluo-NGF *versus* fluo-proNGF Axonal Transport

**Figure 2:**
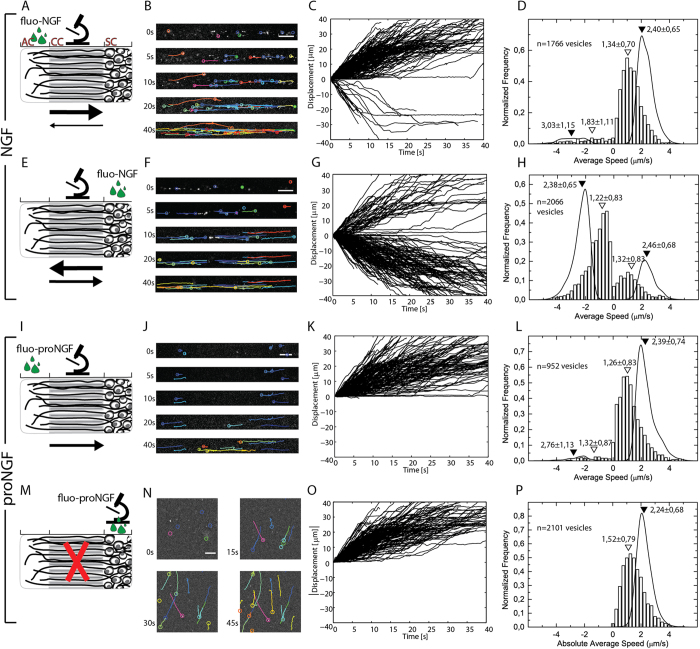
Live axonal transport of fluo-NGF and fluo-proNGF. (**A,E,I,M**) Schematic picture of the microfluidic device where, in the uppermost panel, the axon compartment (AC), the channel compartment (CC), and the soma compartment (SC) are indicated. The green droplets represent NT administration; a stylized microscope indicates the compartment in which fluorescence acquisition is performed; arrows of different dimensions schematize the direction and amount of detected moving vesicles. (**B,F,J,N**) Representative images of the time lapse videos of moving vesicles travelling through the axon. Each coloured line represents a single vesicle trajectory. (**C,G,K,O**) Displacement vs time plot of 200 representative vesicles. (**D,H,L,P**) Bars: average speed distribution of moving vesicles. Lines: distribution of speed during active movement. Empty triangles indicate the mean of vesicles average speed while filled triangles indicate the average speed during active movements; uncertainties are standard deviations. The number of acquired trajectories is reported in each panel. Distributions with areas normalized to 1; in (**C,D,G,H,K,L**), positive and negative displacements or speeds refer to retrograde and anterograde movements respectively, in the configuration described in M-P only the absolute value of displacements and speeds could be determined.

Purified fluorolabeled proNGF and NGF were employed for axonal transport studies in living DRG neurons, cultured in a microfluidic chamber divided in three compartments^29^: a soma compartment (SC), where neurons are plated, a channel compartment (CC), where neurons extend their axons through micrometer channels, and an axon compartment (AC) where neurons spread their axonal tips (Fig. 2A,E,I,M). Each experiment was performed by administering the NT at 2 nM concentration in the AC or in the SC and measuring the axonal transport of the protein by epi-fluorescence microscopy and single particle tracking of fluorescent vesicles.

First, fluo-NGF was administered to the AC or to the SC and the vesicles trafficking was measured in the CC (Fig. 2A,E). In both cases, fluo-NGF filled vesicles started to move about 20 minutes after NT administration. Fluo-NGF vesicles were found to move not only when NGF was delivered to the AC, from the axon tip to the cell soma (retrograde movement), but also when delivered to SC, in the opposite direction (anterograde movement). Typical recorded trajectories are represented in Fig. 2B, F. Most of the fluo-NGF vesicles move with a stop-and-go dynamics, as previously reported^17^, characterized by variable pausing times (Fig. 2C,G). On the whole, traveling vesicles spend about 55% of their time moving (see also Supplementary Fig. 2 and Supplementary Information). Labelled vesicles show a wide distribution of average speeds, when fluo-NGF is administered either at the AC or at the SC (Fig. 2D,H; positive and negative velocities refer to retrograde and anterograde movements, respectively, if not specified otherwise; see also Supplementary Table 1). Notably, in the case of AC-administered fluo-NGF, also anterogradely moving vesicles have been observed, about 10 minutes after the first retrogradely moving vesicle was measured. The speed distribution during the active phase of the overall movement, evaluated by separating the “stop” and “go” parts of the trajectories, is shown as solid lines in Fig. 2D,H. The high number of trajectories acquired with SC-applied fluo-NGF allows to conclude that the anterograde transport is slightly, but significantly (p < 0.05, Dunns Test), slower than the retrograde transport. On the contrary, the low number of anterograde moving vesicles did not allow this comparison in the case of AC-applied fluo-NGF. The retrograde and anterograde average velocities measured by administering fluo-NGF to the AC or the SC are the same within uncertainties (see also Supplementary Table 1).

In the case of fluo-NGF applied to the AC, the anterograde population of moving vesicles increases during the acquisition, with an average value of 9% (Supplementary Fig. 3A). This anterograde movement is different from the short anterograde displacement seen during vesicle retrograde transport, as frequently observed here and previously reported^17^, and persists for large displacements (up to about 100 μm, which represents the length of the field of view) and for long times (more than 60 sec). Furthermore, when fluo-NGF is applied to the SC, about 20 minutes after the appearance of the first anterograde-moving vesicles, we observe the appearance of a robust transport back to the SC. In this case, the number of vesicles transported back (to the SC), after they have had the chance of an outward movement, is significantly higher than that (from SC to AC) observed for AC-administered NGF (25% of the cases compared to 9%) and, once started, this retrograde flux to the soma persists quite constantly for hours (Supplementary Fig. 3B).

Next, proNGF vesicular trafficking was examined under the same experimental conditions as above. Fluo-proNGF was administered to the AC or to the SC (Fig. 2I,M). We found that a retrograde flux of fluo-proNGF vesicles moving from the AC to the SC can be recorded (Fig. 2J), although this is first visible after a longer lag time compared to that of fluo-NGF (starting about 35 minutes after NTs administration). Fluo-proNGF moving vesicles display the same characteristic stop and go movement seen with fluo-NGF (Fig. 2K,O). Vesicles exhibit similar distributions of average speeds and speeds during active movement, compared to the same experiment conducted with mature fluo-NGF. Strikingly, unlike fluo-NGF, when fluo-proNGF was administered to the SC we observed no significant anterograde transport through the axons (Fig. 2M); however, as a proof of neurotrophin internalization, vesicles trafficking with directed motion inside axons in the SC was observed (Fig. 2N–P; note that in this case a polarity to the movement couldn’t be assigned, hence only positive speed is reported, by convention). For comparison, similar measurements for SC applied fluo-NGF are reported in Supplementary Fig. 4. Nevertheless, when fluo-proNGF was given to the AC, a small number of anterogradely moving vesicles (less than 5%), moving just for short displacements, was observed (the maximum anterograde displacement observed is 25 μm). These movements can be confidently attributed to little steps backwards while the vesicle moves retrogradely.

As TrkA and p75 are the main receptors for NGF and proNGF, we investigated by immunofluorescence which of the two receptors mediated the intracellular trafficking of NGF and proNGF. The vast majority of fluo-NGF particles are associated with TrkA receptors; only a subset of them associates with p75, most prominently at the axonal level. Conversely, fluo-proNGF associates with both TrkA and P75 receptors. Notably, we found that when fluo-proNGF is present, p75 also colocalizes with TrkA, and that fluo-proNGF particles are associated with p75/TrkA positive puncta (Supplementary Fig. 5).

### Different proNGF and NGF Content inside the transport Vesicles

**Figure 3:**
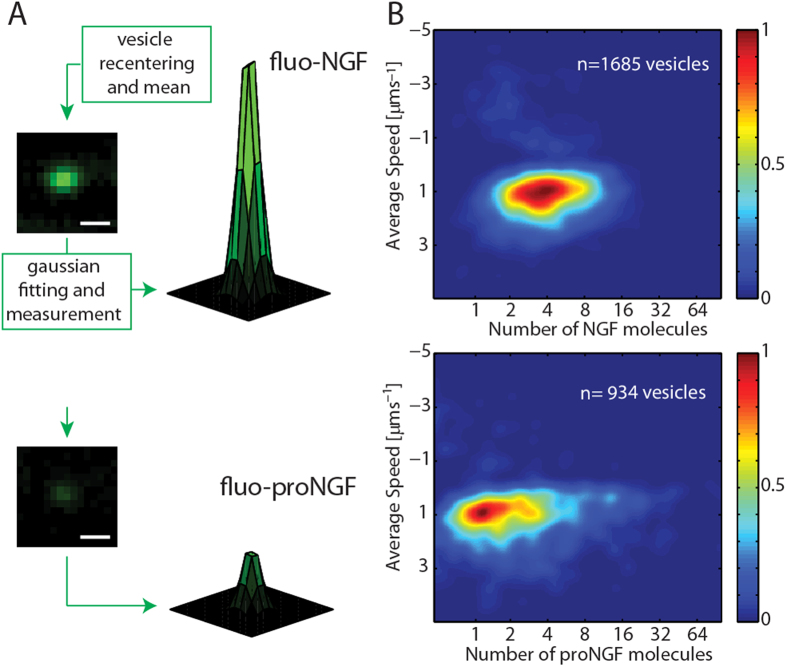
Quantification of NTs number carried by each vesicle. (**A**) Schematization of NT number analysis for both fluo-NGF and fluo-proNGF. A representative re-centered vesicle intensity profile with the corresponding Gaussian fit is reported for both NTs. Scale bar 1μm. (**B**) 2D histograms of NT number carried by vesicles *vs* average speed. The number of analyzed vesicles is reported in each panel.

Visual inspection of the experimental acquisitions of fluorescent NGF and proNGF trafficking (Supplementary Video 1 and 2) suggests that the fluorescent intensity of trafficking vesicles are substantially different in the two cases, despite the identical labelling site and stoichiometry for the two proteins. Two representative fluo-NGF and fluo-proNGF labelled vesicles are presented in Fig. 3A. We quantitatively analyze the different number of NTs transported by a single vesicle in the two cases, thanks to the controlled 1:1 fluorophore:NT-monomer stoichiometry ensured by the labelling strategy. To this end, the mean fluorescence intensity from each vesicle was quantified and normalized to the reference intensities measured from immobilized single fluorophores (see Fig. 3A and Supplementary Methods for details). The measured number of NT dimers, each carrying two fluorophore molecules, is then used to produce a 2D histogram in which the number of NTs is presented *versus* its average speed in Fig. 3B. The position on the Y axis confirms that the average speed of the NGF and proNGF vesicles is the same. The X axis shows that fluo-NGF vesicles carry a range of NT molecules, spanning from 2 to 8 (full width at half maximum) with a mode of 4 dimers per vesicle, while fluo-proNGF vesicles mostly contain a lower number of NTs, from 1 to 4 proNGF dimers with a mode value of 1 dimer per vesicle (see also Supplementary Fig. 6).

### NGF competes with proNGF for axonal transport

**Figure 4:**
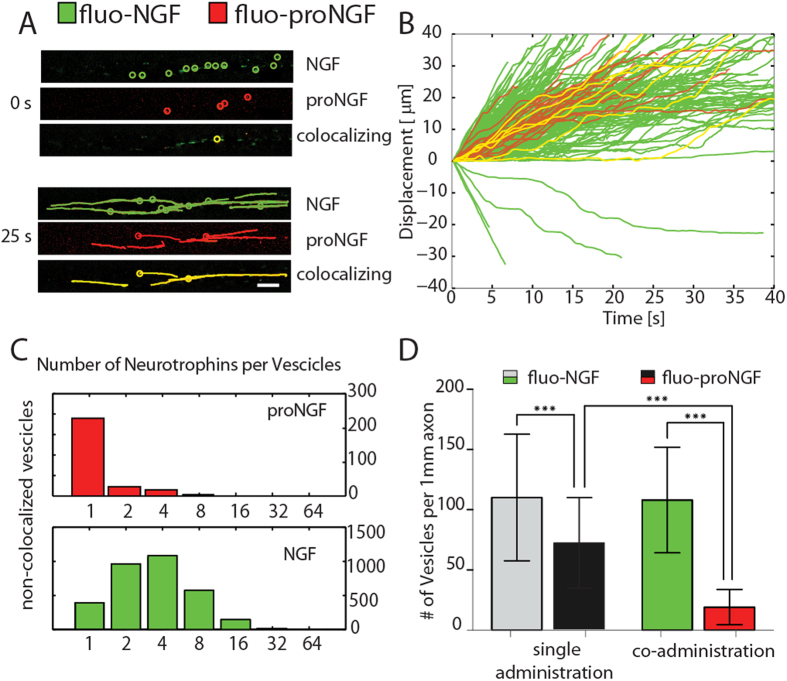
Co-administration of fluo-NGF and fluo-proNGF to the axon compartment. (**A**) Representative images of the time lapse videos of moving vesicles travelling through the axon, at time 0s and after 25 sec; colocalizing (yellow) indicate the vesicles with both NGF and proNGF. (**B**) Displacement *vs* time for a representative 10% of all observed vesicles. The colour code is the same as in panel A. (**C**) Histograms for the number of fluo-proNGF and fluo-NGF per vesicle in non-colocalizing (proNGF red and NGF green) vesicles. (**D**) Histogram representing mean ± SD of the number of vesicles observed per 1mm of axons in the single administration of fluo-NGF and fluo-proNGF case (light grey and dark grey bars) and in case of coadministration (green and red bars). Dunn’s Multiple Comparison Test was performed, ***indicates p < 0.001.

NGF and proNGF coexist *in-vivo*, but a direct observation of simultaneous axonal transport is still lacking. In order to study the simultaneous axonal transport of proNGF *vs* NGF, NTs were conjugated to spectrally distinct probes by using two different CoA-fluorophore substrates: CoA-Alexa647 and CoA-Alexa488 respectively (see Materials and Methods). fluo-NGF and fluo-proNGF (Fig. 4A *Top*) were co-administered, at equimolar (2 nM) concentration, to the AC. A typical dual-colour acquisition of the trajectories recorded in the CC is represented in Fig. 4A, with green and red colours representing putative NGF and proNGF vesicles, respectively. Notably, a population of vesicles carrying both fluorophores, represented in yellow in Fig. 4A,B, was registered.

The velocity of dual-labelled vesicles is not significantly different from that of vesicles carrying the individual NTs (Fig. 4B), and from the velocities registered for the individual administrations of proNGF and NGF (Supplementary Fig. 7); however, we noticed that the number of anterogradely moving vesicles of NGF is decreased to ~3% of the total in this case (Supplementary Fig. 8). Then, the number of neurotrophin molecules carried by vesicles was determined (Fig. 4C). The distributions of fluo-NGF is quite similar to that observed after a single administration, while, interestingly, the number of proNGF dimers was markedly reduced at 1 in the vast majority of cases, demonstrating a great preference of vesicles to transport mature NGF instead of the precursor form (Fig. 4C and Supplementary Fig. 6). Similar conclusions can be drawn by measuring the vesicle fluxes with fluo-NGF and fluo-proNGF, given separately or simultaneously at the AC (Fig. 4D). In the case of single administrations, the number of moving vesicles per 1 mm of axon is 110 ± 42 and 72 ± 38 (mean ± SD) for fluo-NGF and for fluo-proNGF respectively. This indicates that, when administered alone and at the same concentration, proNGF vesicles flux is ~65% of the NGF one. The fluo-proNGF flux dramatically drops down to 19 ± 14 vesicles per 1 mm of axon (i.e. ~18% of NGF flux) when the two NT forms are given together, while fluo-NGF vesicles flux is similar to that observed after the individual NT administration. Actually, the real proNGF flux could be even lower than that observed due to the possibility of a small fraction of fluo-proNGF being cleaved to its mature counterpart during the acquisition time window (Supplementary Fig. 9). Thus, upon co-administration we observed for proNGF a reduction both in the flux of vesicles and in the number of neurotrophins per vesicle that might stem from competition with NGF or from signaling events caused by NGF and proNGF simultaneous administration (see Discussion for more details)^7^.

## Discussion

The axonal transport of NTs is a crucial aspect of their mode of action in the nervous system. In this context, despite the fact that proNGF has a receptor binding profile distinct from that of NGF^30^,^31^, very little is known on how this reflects in the transport properties of proNGF *versus* those of mature NGF. We address this issue through a novel strategy to fluorolabel purified recombinant precursor and mature NTs with small organic dyes, exploiting a recently reported chemical-tag based NT labeling^24^. The method has several advantages over previously used NT labelling procedures: i) the precise control of stoichiometry (1:1 NT-monomer:probe) and site (C-terminus) of fluorophore conjugation; ii) the complete purification of the labelled species from unlabelled counterparts as well as from free fluorophores (Fig. 1F); iii) the versatility of the used tags, which can be functionalized by virtually any kind of probe, e.g. biotin or fluorophores (Fig. 1E,F), that can be carried by coA substrates; iv) the possibility to simultaneously study two “homologue” molecules with orthogonal fluorolabels (Fig. 4).

The obtained fluo-NGF and fluo-proNGF probes allowed here the first comparative imaging and tracking of proNGF and NGF axonal transport in living DRG neurons by single molecule fluorescence microscopy. Results unambiguously show that proNGF is retrogradely transported inside vesicles like its mature counterpart (Figs 2, 3, 4). proNGF displays the same stop-and-go movements previously reported for NGF and an average speed distribution similar to that of NGF. These data suggest that the endocytic pathway and related engaged molecular motors are conserved between proNGF and NGF. Beside these similarities, the axonal transport of these two proteins differs from each other in several significant aspects.

First, proNGF vesicles move exclusively from the axon tip to the cell soma of neurons, while NGF vesicles exhibit movements in both directions (Fig. 2). While a retrograde trafficking has been paradigmatically described for mature NGF^10^, a bidirectional NGF transport has been previously described in neurite-like processes of PC12 cells, either directly using Cy3.5-NGF^13^ or indirectly by studying TrkA trafficking^32^,^33^. It should be considered, however, that neurites in PC12 do not have comparable biochemical and functional features to DRG ones. In our experiments, anterograde (centrifugal) movement when NGF is administered at the AC accounts for ~10% of the total vesicles analyzed (Fig. 2C), consistently to what reported, for tracked quantum-dot conjugated NGF^17^, in the same neurons. The latter study, however, did not quantitatively analyze the 10% anterograde trajectories and did not study proNGF. Considering NGF axonal transport as purely retrograde was therefore previously suggested^17^. Nevertheless, we believe this smaller population of anterogradely moving vesicles containing NGF should not be neglected because: 1) it is increased (up to ~75%) when NGF is administered to the SC; 2) it is not observed with fluo-proNGF applied to the AC nor to the SC; 3) it is decreased to ~3% upon the simultaneous administration of proNGF (Supplementary Fig. 8). These data point to anterograde centrifugal movement as a specific feature of NGF but not of proNGF, with the latter interfering with the transport of the former. While these considerations strengthen the idea that NGF and proNGF are indeed two different signalling molecules, the biological significance of the observed anterograde movement remains to be established. It could indeed be that this is a key feature of survival/differentiation responses; however, it could also be that in our experimental conditions NGF in the SC passively exploits the well-known Trk anterograde transport^34^,^35^ and that p75 does not support proNGF anterograde movement in this cellular model.

Secondly, proNGF and NGF vesicles contain a different number of molecules per vesicle (Fig. 3). Data demonstrate that each vesicle mostly hosts 1 or 2 proNGF and between 2 and 8 NGF dimers, when NTs are administered separately, while proNGF number is drastically decreased to one, when NTs are co-administered, indicating that NGF provides a veto or competing signal for proNGF internalization and transport. This finding does not appear to match with the recently proposed idea that the typical functional signaling endosome consists of a single NT dimer bound to a single pair of TrkA receptors^17^. Rather, it is consistent with a model that envisions the existence of a larger ligand-receptor complex per vesicle, in which a higher number of NTs are clustered together. This discrepancy could arise from the largely different steric hindrance of the two fluorolabels used in the two studies. In fact, Cui *et al.* used Quantum Dots (QD)-conjugated NGF (each QD putatively coupled to a single NGF dimer)^17^. As QD volume is up to 70 times that of NGF (Supplementary Fig. 10), the QD-NGF conjugate might impair NGF clustering^14^, thus artifactually preventing the simultaneous internalization of several NGF molecules. This would lead to a decrease in the observed number of QD-NGF internalized and transported per vesicle. Conversely, using much smaller organic dyes to fluorolabel NGF, as done here, might allow accommodating a physiologically higher number of clustered NGF molecules that could easily find room in the same vesicle. In this context, it is worth mentioning that, using ^125^I labelled NGF (^125^I -NGF), Campenot and coworkers^36^ argued that the total amount of ^125^I -NGF transported to the soma would require two orders of magnitude more vesicles with respect to what measured by Cui *et al.*^17^, if such vesicles contained only one NGF molecule.

Finally, since in physiological contexts NGF and proNGF coexist, the two forms of the NT were co-administrated to the AC (Fig. 4), highlighting a competitive mechanism between them (proNGF flux and number of molecules per vesicle was strongly inhibited and reduced by NGF co-administration). Taking these data together with immunofluorescence data (Supplementary Figure 5), we shall propose two possible mechanisms for the observed competition, which are interestingly not mutually exclusive. First, there could be simply a competition for the receptors available on the plasma membrane of the axon tip. Immunofluorescence analysis shows that the vast majority of fluo-NGF particles are associated with TrkA receptors, whereas only a subset of them associate with P75, and fluo-proNGF particles associate with both TrkA and P75 receptors. Thus, the two neurotrophin forms would compete for TrkA binding. The nature of proNGF - TrkA interaction is still elusive and likely occurs with less affinity than NGF-TrkA interaction^37^; in any case, free TrkA could be necessary for proNGF internalization, and sequestration of free TrkA by NGF could affect proNGF transport so that the only possibility is that it enters via p75; the latter has well recognized slower internalization rate compared to TrkA in peripheral neurons^12^, and this could finally result in a reduction of fluo-proNGF transport compared to that of fluo-NGF in our observation-time window. However, NGF/proNGF reciprocal signalling per se may alter endocytosis and transport. Thus another possibility is that NGF, rather than being a simple competitor of proNGF for TrkA receptor binding, activates some signalling pathway that prevents proNGF to internalize. It is useful to recall here that different proNGF/NGF mixtures exert a signalling profile that is different from that raised separately by the two neurotrophins and that depends on the ratio between the two proteins^7^. In any case, whatever the competition mechanism, the most important finding emerging from these data is that proNGF retrograde signalling, which is likely to be pro-apoptotic, can emerge more when an NGF trophic signalling is absent. One could therefore expect that NGF pro-survival signalling is dominant with respect to proNGF one. Alterations in the NGF to proNGF ratios might have direct consequences in the transport fluxes and hence in the availability of the two NTs in a competitive way, adding a new functional consequence to the emerging importance of the homeostatic regulation of proNGF to NGF metabolism^9^,^38^.

From a general perspective, then, the labeling platform proposed here could also be exploited to achieve the purification of vesicles carrying NTs and/or the respective proNTs, leading to a proteomic characterization of NT signalling endosomes. This would lately allow to discover whether the molecular composition and identity of traffic and signalling endosomes are dependent on the transported cargo^39^,^40^. Furthermore, we envisage the possibility of using our approach for the setup of chemical or functional genomic screening assays for compounds or genes that regulate the velocity of NTs transport.

## Materials and Methods

### Expression, purification and refolding of proNGF-tag and NGF-tag

Human proNGF cDNA cloned in pET11 vector^22^ was used as template. The cDNA coding sequences of each tag was inserted into proNGF, by using an insertional mutagenesis procedure adapted from the site-directed mutagenesis method^24^. wt and tagged proNGF proteins were expressed in *E. coli* and purified using a modified protocol^22^ from the previously published method^28^.

### Labelling of tagged proNGF and NGF

10 μg of proNGF-YBBR or NGF-YBBR were incubated for 30 minutes at 37 °C in a thermomixer at 300 rpm with a reaction mix (10 mM MgCl_2_, 10 μM CoA-alexa488/CoA-alexa 647 or CoA-biotin and 2 μM Sfp Synthase (SfpS) (New England Biolabs), in phosphate buffer up to 250 μl final volume. The synthesis of CoA-biotin and CoA-fluorophore conjugates has been performed as previously described^37^.

### PC12, TF1 and DRG cultures

PC12 (ATCC, CRL-1721) cells were maintained in a humidified atmosphere at 37 °C, 5% CO_2_ in RPMI1640 medium supplemented with 10% horse serum, 5% fetal bovine serum and 1% penicillin/streptomycin (Gibco). TF1 cells were cultured in RPMI 1640 medium, 10% fetal bovine serum and 1% penicillin/streptomycin (Gibco), supplemented with 2 ng/ml recombinant human GM-CSF (R&D Systems Inc.). The TF1 proliferation assay was performed in 96 wells microtiter plates by incubating 15,000 cells per well in the presence of increasing doses of either wild type hNGF and NGF-YBBR or NGF-A4, ranging between 5 and 50,000 pg/ml.

Rat Dorsal Root Ganglion Neurons (R-EDRG-515 AMP, Lonza) were maintained in a humidified atmosphere at 37 °C, 5% CO_2_ in Primary Neuron Basal medium (PNBM, Lonza) supplemented with L-glutamine, NSF-1 (2% final concentration) and antibiotics, following the manufacturer’s instructions. For DRG neurons survival the media was supplemented by 100 ng/ml of NGF and was replaced every 4–5 days with a pre-warmed fresh one, being careful to always leave the main channels filled. When neurons were plated in microfluidic devices, a NGF gradient (obtained leaving the SC with 50 ng/ml of NGF, and the AC in the presence of 100 ng/ml) was used to induce axons to grow in the CC and to reach the AC. DIV8-15 cultures of DRG neurons were used in all experiments: within this range, we did not detect significant changes in the NT vesicles transport. In all the transport experiments, the fluorescent NT has been administered in fresh media (not supplemented with NGF) by removing from the administration compartment all the pre-existing media.

### Western Blot

In order to quantify proNGF and NGF biotinylation reaction yields, 2 μl of all NGF/proNGF biotinylation reactions were treated under denaturing conditions (100 °C, 8 minutes in 2X Laemmli Sample Buffer), loaded on two gels (1 μl for each gel) and electrotransferred to two PVDF membranes respectively. These were blocked in TBST + 5% w/v BSA, then one of them was blotted with anti-NGF antibody (sc-549, Santa Cruz Biotechnology) (1:2000), while the other one was incubated with HRP-conjugated streptavidin (Zymed®) 1:10000 diluted in blocking solution.

To study the signal transduction effectors, PC12 cells were cultured in P100 Petri dish to reach confluence, starved o.n. in a serum-free medium and then incubated with native NGF, tagged NGF, biot-NGF and fluo-NGF (150 ng/ml) at 37 °C. After 15 min cells were washed in ice-cold PBS and lysed in RIPA buffer supplemented with proteases and phosphatases inhibitors. 50 μg of each clarified lysate were loaded on a gel and electrotransferred to PVDF membranes. These were first blotted using the antibody anti-Phospho-PLCγ1, anti-Phospho-p44/42 MAPK and anti-Phospho-Akt. The primary antibody was detected by using an anti-mouse or rabbit secondary antibody HRP-conjugated.

### Microscopy

All microscopy measurements have been conducted in a wide field microscope (Leica DM6000, equipped with a 4-laser TIRF-AM module) at 37 °C, 5% CO_2_. Transmitted light imaging has been performed in differential interference contrast (DIC) configuration. For epi-fluorescence microscopy 488 and 633 solid state lasers have been used to excite Alexa488 and Alexa647 respectively.

### Single Particle Tracking and Number analysis

Single Particle Tracking analysis was performed by custom made Matlab scripts following the approach previously described^41^. Colocalizing trajectories were estimated by visual inspections of extracted trajectories in the green and red fluorescence channels, after an initial automatic selection based on the average time and positions of each trajectory in each microfluidic channel.

## Additional Information

**How to cite this article**: De Nadai, T. *et al.* Precursor and mature NGF live tracking: one *versus* many at a time in the axons. *Sci. Rep.* **6**, 20272; doi: 10.1038/srep20272 (2016).

## Supplementary Material

Supplementary Information

Supplementary Video 1

Supplementary Video 2
