# Corrigendum: Precursor and mature NGF live tracking: one *versus* many at a time in the axons

**DOI:** 10.1038/srep23308

**Published:** 2016-03-30

**Authors:** Teresa De Nadai, Laura Marchetti, Carmine Di Rienzo, Mariantonietta Calvello, Giovanni Signore, Pierluigi Di Matteo, Francesco Gobbo, Sabrina Turturro, Sandro Meucci, Alessandro Viegi, Fabio Beltram, Stefano Luin, Antonino Cattaneo

Scientific Reports 6: Article number: 20272;10.1038/srep20272 published online: 02 01 2016; updated: 03 30 2016

This Article contains errors in Affiliation 3. The correct affiliation is listed below:

Center for Nanotechnology Innovation @NEST, Istituto Italiano di Tecnologia, Piazza San Silvestro 12-56127 Pisa, Italy.

