# Supplementary material for: Precursor and mature NGF live tracking: one versus many at a time in the axons
Source: Sci Rep. 2016 Feb 1;6:20272. doi: 10.1038/srep20272 (PMC4735336; doi:10.1038/srep20272)
Supplement: Supplementary Information [file srep20272-s1.pdf]

Supplementary Information

Precursor and mature NGF live tracking: one *versus* many at a time in the axons.

Teresa De Nadai [a], Laura Marchetti [a,b], Carmine Di Rienzo [b,c], Mariantonietta Calvello [a], Giovanni Signore [c], Pierluigi Di Matteo [a] , Francesco Gobbo [a] , Sabrina Turturro [d] ,Sandro Meucci [b,c], Alessandro Viegi [a], Fabio Beltram [b,c], Stefano Luin [b], and Antonino Cattaneo [a]*.

[a]. BioSNS Laboratory, Scuola Normale Superiore and Istituto di Neuroscienze - CNR, Pisa, Italy,
[b]. NEST, Scuola Normale Superiore and Istituto Nanoscienze – CNR, Pisa, Italy,
[c]. IIT@NEST, Center for Nanotechnology Innovation, Pisa, Italy,
[d]. EBRI, European Brain Research Institute, Rome, Italy

Corresponding Author:

* antonino.cattaneo@sns.it

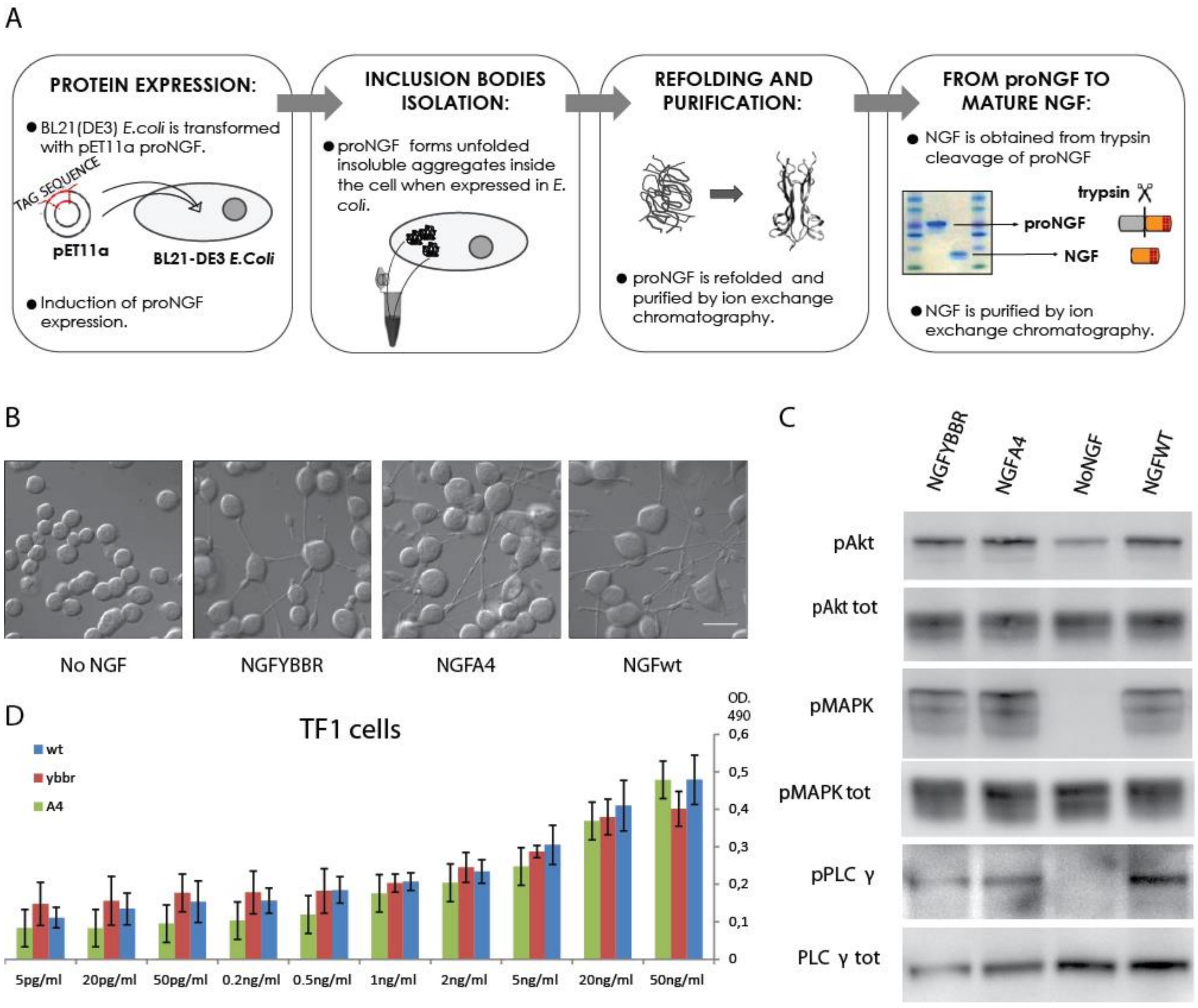

Supplementary Fig. 1. Production and functional validation of tagged neurotrophins (A) Schematization of the different steps of proNGF and NGF synthesis and purification protocol. (B) Typical DIC images obtained performing the differentiation assay in PC12 cells using equimolar amounts of wt NGF, NGF-YBBR and NGF-A4. Untreated cells are represented as control. Scale bars represent 20 µm. (C) Western blot analysis of phosphorylated Akt (pAkt), phosphorylated Erk 1/2 (pErk1/2) and phosphorylated PLCγ (pPLCγ) protein levels in PC12 cells in response to wt NGF, NGF-YBBR and NGF-A4, compared to the same obtained for untreated cells (No NGF); the signal of the total respective signaling effector is the loading control. (D) TF1 factor-dependent human erythroleukemic cells proliferation assay performed administrating wt NGF, NGF-YBBR and NGF-A4.

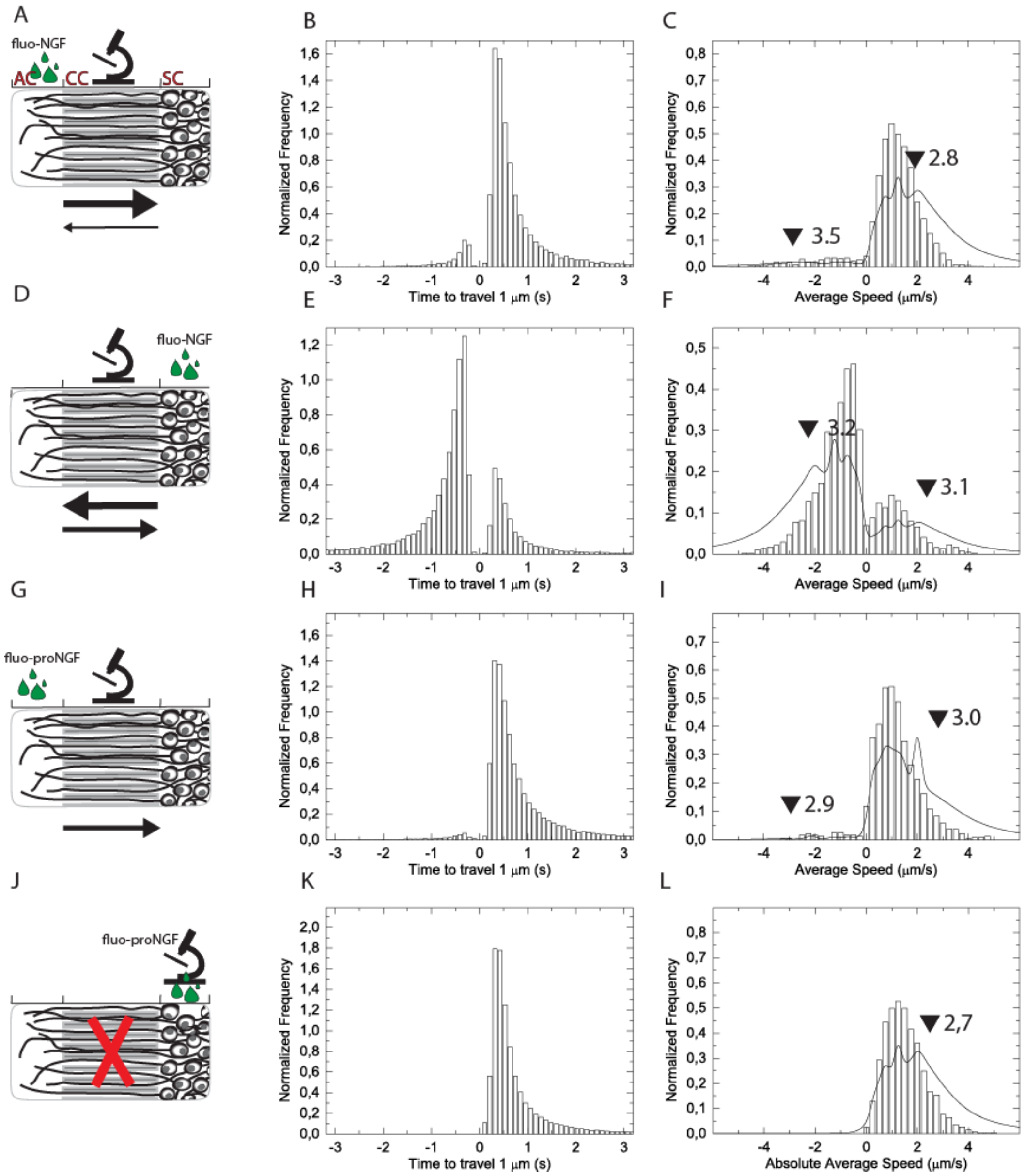

Supplementary Fig. 2. Live axonal transport of fluo-NGF and fluo-proNGF: determination of speed distributions in 1 μm steps of measured trajectories; this kind of distribution will weigh more the speed during active movements. (A,D,G,J) Schematic picture of the microfluidic device; green droplets, stylized microscope and arrows have the same meaning as in Fig.2. (B,E,H,K) Bars: histograms of times required for the vesicles to move 1 μm. (C,F,I,L) Bars: average speed distribution of moving endosomes (same data of Fig. 2, for comparison). Lines: distribution of speeds for steps of 1 μm, obtained by the corresponding time distributions in panels B, E, H, K. Distributions with areas normalized to 1; in B, C, E, F, H, I, positive

(negative) times or speeds refer to retrograde (anterograde) movements, in the configuration described in J-L only the absolute value of displacements and speeds could be determined.

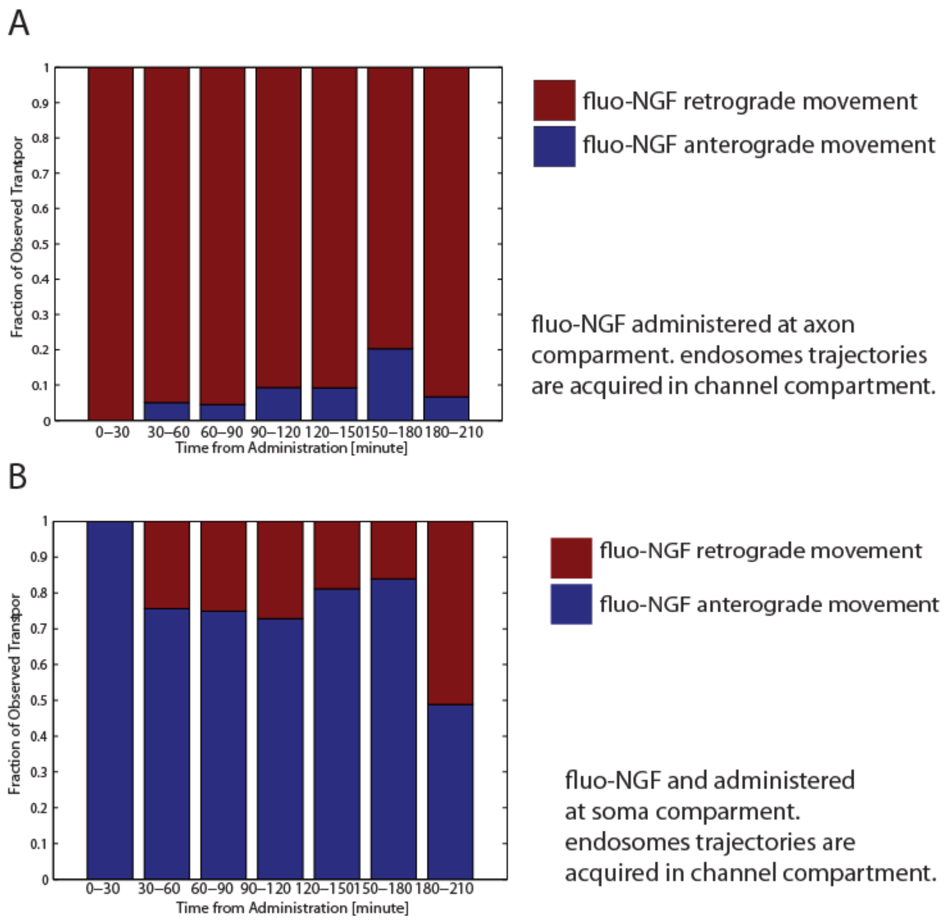

Supplementary Fig. 3 Fraction of retrograde and anterograde trajectories vs time. (A) Bar graph of the fraction of anterograde *vs* retrograde recorded trajectories when fluo-NGF is administered to the AC and time lapse imaging performed in the CC. (B) Same as (A) when fluo-NGF is administered to the SC and time lapse imaging performed in the CC.

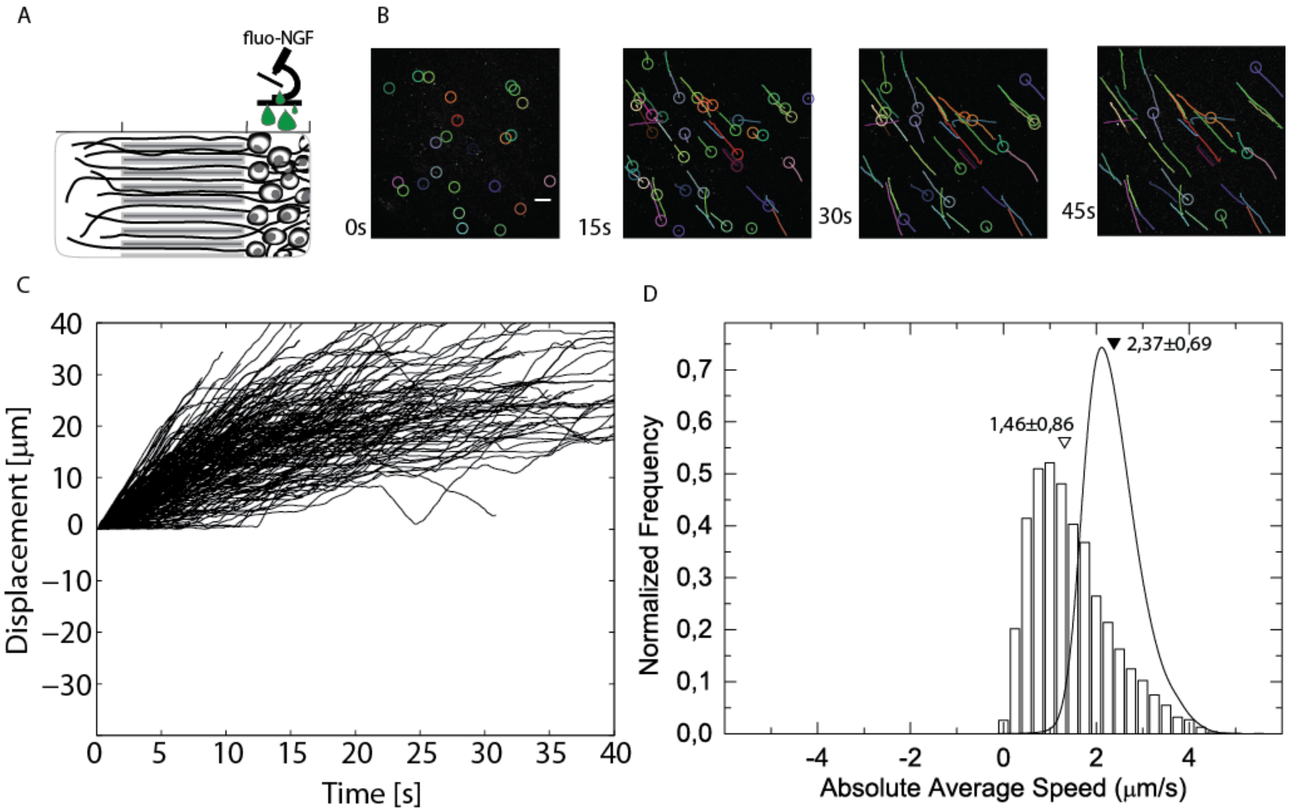

Supplementary Fig. 4. Live axonal transport of fluo-NGF outside the microfluidic channels. (A) Schematic drawing of the microfluidic device. The green droplets represent fluo-NGF administration compartment; time lapse imaging is performed in SC; (B) Representative images of the time lapse videos of moving vesicles travelling through the axon. Each colored line represents a single endosome trajectory. (C) Displacement vs time of 200 representative endosomes. (D) Bars: average speed distribution of moving endosomes. Lines: distribution of speed during active movement. Empty and filled triangles indicate the mean of endosomes speed and the mean speed during active movements respectively; uncertainties: standard deviation. The number of acquired trajectories is n=6316. Distributions with areas normalized to 1; in this configuration, only the absolute value of displacements and speeds could be determined.

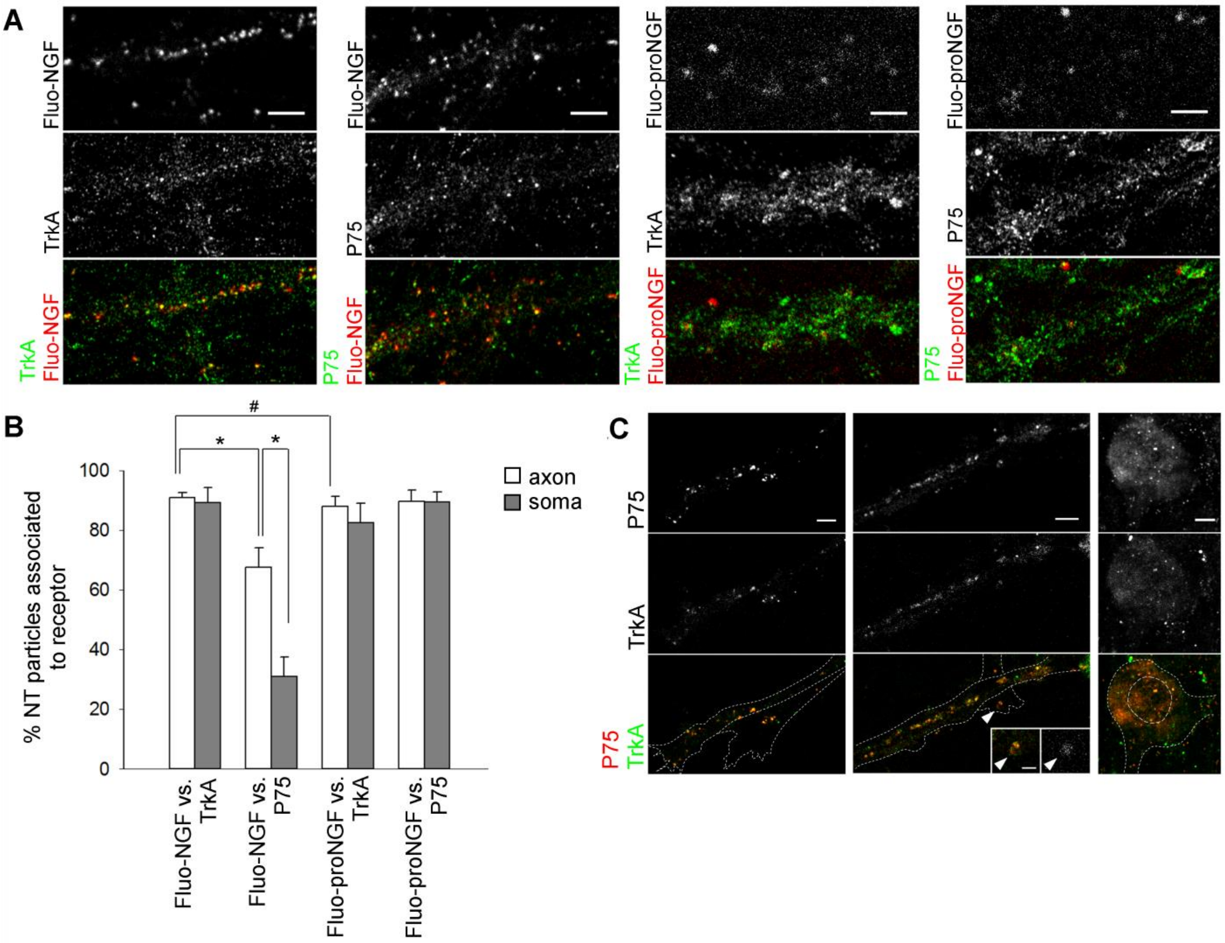

Supplementary Fig. 5. (A) Colocalization of neurotrophins and their receptors. Immunofluorescence (IF) analysis for NT association to TrkA and p75. The vast majority of fluo-NGF particles are associated with TrkA receptors, whereas only a subset of them associate with p75. Conversely, fluo-proNGF associates with both TrkA and p75 receptors. First row, fluo-(pro)NGF fluorescence; second row, IF against TrkA or p75, as indicated; third row, merge image. Scale bar 5 μm. (B) Quantification of associated NT-receptor pairs as in A. Fluo-NGF strongly interacts with TrkA both along the axon and in the soma, as fluo-proNGF with p75. A smaller proportion of fluo-NGF is associated to P75, and most of NGF particles in the soma are devoid of p75. # P<0,01, and * P<0.001, two-tailed Student's t-test. Bars represent mean ± standard deviation. (C) Double immunofluorescence for TrkA and p75 in fluo-proNGF treated neurons. A great number of TrkA particles co-localized with p75 puncta, preferentially at axon termini and along the axon, and, to a

lesser extent, within the soma. Fluo-proNGF can be detected at double-positive puncta (inset, arrowhead). First row, p75 IF; second row, TrkA IF; third row, merge image. Scale bar 5 μm, inset 2 μm. The cell profile is indicated with dotted lines, whereas broken line indicates nucleus position, as evaluated by transmission and DAPI channels, respectively.

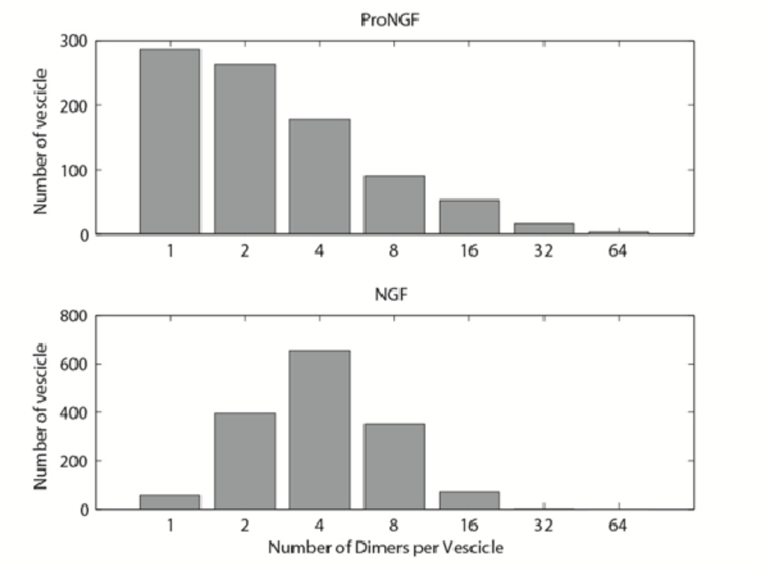

Supplementary Fig. 6. Histograms for the numbers of fluo-proNGF and fluo-NGF per vesicle in single administration experiments.

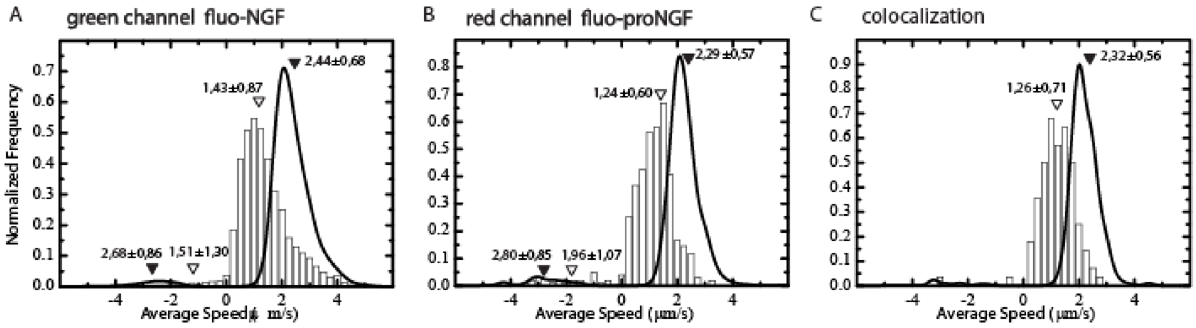

Supplementary Fig. 7. Vesicle dynamics upon co-administration of fluo-proNGF and fluo-NGF. Average speed distribution of moving endosomes (bars) and distribution of speeds during active movement (lines) for vesicles containing fluo-NGF (A), fluo-proNGF (B) and both of them (C).

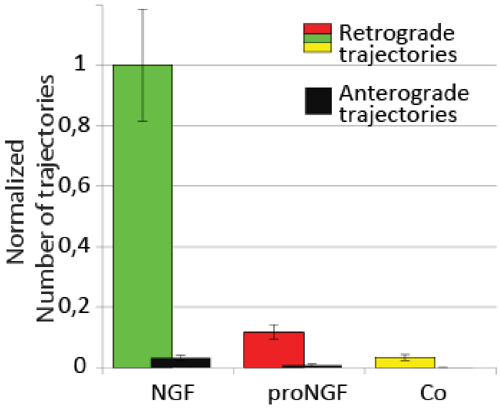

Supplementary Fig. 8. The number of moving proNGF vesicles and of anterograde moving NGF vesicles decreases upon co-administration of equimolar quantities of NGF and proNGF. Bars: total number of trajectories per channel measured in the CC for fluo-NGF vesicles (NGF), fluo-proNGF (proNGF) vesicles and for vesicles containing both NTs (Co). The number of fluo-NGF retrograde-moving vesicles is assumed as a reference and normalized to one. The error bars represent the SD between two different experiments.

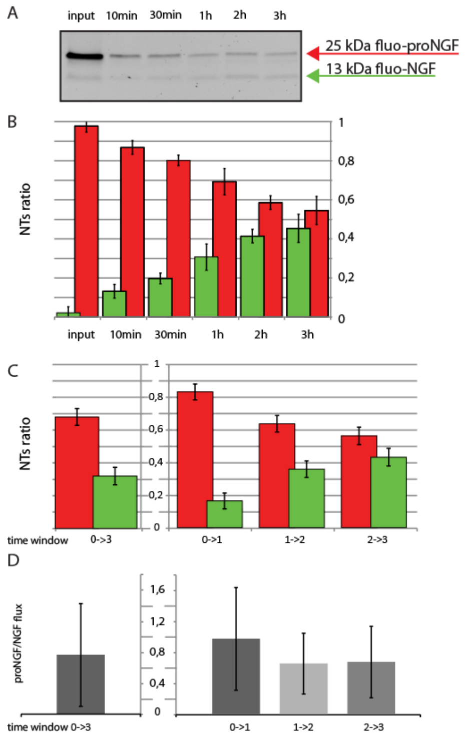

Supplementary Fig. 9. Precursor NTs vesicles contain the full length proNGF. Fluo-proNGF is represented in red, and fluo-NGF in green. (A) SDS-PAGE of PC12 cells lysate after fluo-proNGF administration. 10 min, 30 min, 1h, 2h and 3h represent the period of incubation. The input represents the signal of fluo proNGF spiked into untreated PC12 cell lysate. (B) Histogram representing the percentage of precursor and mature fluorescent neurotrophins from two different experiments as the one represented in panel A (C) Quantification of the average percentage of precursor and mature fluorescent neurotrophins during the first (1h), second (2h) and third (3h) hour of incubation. Data derive from the average of two replicas. (D) Histogram representing the ratio between proNGF and NGF flux during single administration experiments. The error bars of both histograms derive from the standard deviation of the data by standard propagation of uncertainties.

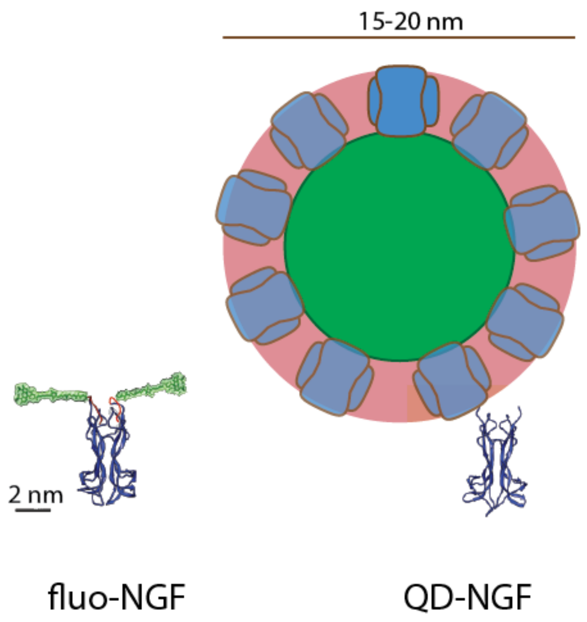

Supplementary Fig. 10. Visual comparison between fluo-NGF and QD-NGF sizes. The two scale bars underline the different dimension of fluorescent NGF labelled with the presented method (*Left*) and one of the methods previously reported in literature[1] (*Right*). In these picture, the impact of the fluorophore on the dimensions of the tagged NGF seems even higher than it is, because the schematic view of NGF and tag structures schematizes only the backbone (with no residuals), while for the Alexa488-maleimide-phosphopantetheinyl the whole structure formula with a linking chain in extended configuration is given.

Supplementary Video 1. Live imaging of fluo-NGF (2nM) containing endosomes in axons of DRG neurons after background subtraction. The video was acquired about 69 min after neurotrophin administration.

Supplementary Video 2. Live imaging of fluo-proNGF (2nM) containing endosomes in axons of DRG neurons after background subtraction. The video was acquired about 86 min after neurotrophin administration.

Retrograde and anterograde average velocities:

		Acquisition chamber	Administration chamber	Retrograde Velocity (μms^{-1})	Standard deviation	Standard error	% of measured trajectories	Antetrograde Velocity (μms^{-1})	Standard deviation	Standard error	% of measured trajectories
NGF		CC	AC	1,34	0,70	0,02	91	1,83	1,11	0,09	9
		CC	SC	1,32	0,83	0,04	25	1,22	0,83	0,02	75
		SC	SC	\\	\\	\\	\\	1,46	0,86	0,01	100
proNGF		SC	SC	\\	\\	\\	\\	1,52	0,79	0,02	100
		CC	AC	1,26	0,83	0,03	95	1,32	0,87	0,13	5
		CC	SC	1,39	0,47	0,23	44	0,75	0,37	0,17	56
NGF and pro NGF	Non colocalized	CC_(pro NGF)	AC	1,24	0,60	0,03	94	1,96	1,07	0,22	6
		CC_(NGF)	AC	1,43	0,87	0,02	97	1,51	1,30	0,13	3
	Colocalized	CC_(pro NGF)	AC	1,26	0,55	0,05	98	1,71	1,83	1,29	2
		CC_(NGF)	AC	1,26	0,71	0,07	99	3,11	Inf	Inf	1

Supplementary Table 1. The retrograde and anterograde average velocities measured in the CC (channel compartment) or in the SC (soma compartment) by administering fluo-NGF and /or fluo-proNGF to the AC (axon compartment) or the SC.

List of mutagenesis primers used for plasmids preparations.

primer name	Sequence (5’→ 3’)
**YBBR-insert1-FW**	G CTC TCT AGA AAG GCT GTG AGA GAT TCT CTT GAA TGA TAA GGA TCC GGC TGC TAA C
**YBBR-insert1-RV**	G TTA GCA GCC GGA TCC TTA TCA TTC AAG AGA ATC TCT CAC AGC CTT TCT AGA GAG C
**YBBR-insert2-FW**	G GCT GTG AGA GAT TCT CTT GAA TTT ATT GCT AGT AAG CTT GCG TGA TAA GGA TCC GGC TGC TAA C
**YBBR-insert2-RV**	G TTA GCA GCC GGA TCC TTA TCA CGC AAG CTT ACT AGC AAT AAA TTC AAG AGA ATC TCT CAC AGC C
**S6-A1-insert1-FW**	G CTC TCT AGA AAG GCT GTG AGA GGA GAT TCT CTT TGA TAA GGA TCC GGC TGC TAA C
**S6-A1-insert1-RV**	G TTA GCA GCC GGA TCC TTA TCA AAG AGA ATC TCC TCT CAC AGC CTT TCT AGA GAG C
**S6-insert2-FW**	CT GTG AGA GGA GAT TCT CTT TCG TGG CTG CTT AGG CTT TTG AAT TGA TAA GGA TCC GGC TGC TAA C
**S6-insert2-RV**	G TTA GCA GCC GGA TCC TTA TCA ATT CAA AAG CCT AAG CAG CCA CGA AAG AGA ATC TCC TCT CAC AG
**A1-insert2-FW**	CT GTG AGA GGA GAT TCT CTT GAT ATG TTG GAG TGG TCT TTG ATG TGA TAA GGA TCC GGC TGC TAA C
**A1-insert2-RV**	G TTA GCA GCC GGA TCC TTA TCA CAT CAA AGA CCA CTC CAA CAT ATC AAG AGA ATC TCC TCT CAC AG
**A4-insert-FW**	CTC TCT AGA AAG GCT GTG AGA GAT TCT CTT GAT ATG TTG GAG TGG TGA TAA GGA TCC GGC TGC
**A4-insert-RV**	GCA GCC GGA TCC TTA TCA CCA CTC CAA CAT ATC AAG AGA ATC TCT CAC AGC CTT TCT AGA GAG
**A4-insert1-FW**	G CTC TCT AGA AAG GCT GTG AGA GAT TCT CTT GAT TGA TAA GGA TCC GGC TGC TAA C
**A4-insert1-RV**	G TTA GCA GCC GGA TCC TTA TCA ATC AAG AGA ATC TCT CAC AGC CTT TCT AGA GAG C
**A4-insert2-FW**	G GCT GTG AGA GAT TCT CTT GAT ATG TTG GAG TGG TGA TAA GGA TCC GGC TGC TAA C
**A4-insert2-RV**	G TTA GCA GCC GGA TCC TTA TCA CCA CTC CAA CAT ATC AAG AGA ATC TCT CAC AGC C

Supplementary Table 2. List of mutagenesis primers used for plasmids preparations.

Supplementary Discussion

proNGF vesicles contain the full length precursor NT

Since the fluorescent label is located at the C-terminus of proNGF and NGF, the question could arise whether the observed proNGF vesicles do contain the full length protein, or, rather, its mature cleavage product. To address this question, a time course of proNGF cleavage upon cells administration was performed, over the three-hours' time range of transport data acquisition. (Supplementary Fig. 9A and 9B). Obtained data indicate that the amount of fluo-NGF generated during the first, second and third hours of incubation is 16%, 36% and 43% respectively (Supplementary Fig.9C). The average over the three hours period of transport acquisition is about 30%, i.e. 70% of administered fluo-proNGF molecules are on average still present during this period. We compared the fluo-proNGF to fluo-NGF cleavage kinetics in the three time windows 0-1, 1-2 and 2-3 hours with the fluo-proNGF/fluo-NGF flux ratio in the same time windows (Supplementary Fig.9D, calculated from Fig. 9B). In the first hour, the measured flux of proNGF and NGF are equal (their ratio is about 1, Supplementary Fig. 9D). If the *bona fide* observed transported proNGF vesicles corresponded to cleaved NGF, one would here observe only a 16% (for the 1 hour average cleavage, Supplementary Fig. 9C) of the observed NGF flux. Thus, we are confident that at least the 70% of vesicles that we observed when proNGF is administered to neurons contains the full length proNGF (Fig. 4 and Supplementary Fig. 9). Moreover, the two species display different transport properties. On purpose we decided not to use one of the cleavage-resistant forms of proNGF so far proposed in literature, because these display different biological outcome from each other[2,3], and because they are not completely resistant to cleavage, as it has been shown by the Neet group[4].

Supplementary Materials and Methods

Plasmids preparation. Human proNGF cDNA cloned in pET11a vector[5] was used as template. The cDNA coding sequences of YBBR (DSLEFIASKLA), A4 (DSLDMLEW), A1 (GDSLDMLEWSLM) and S6

(GDSLSWLLRLLN) tags were inserted into the C-terminus of proNGF, by using an insertional mutagenesis procedure adapted from the site-directed mutagenesis method[6]. Briefly, the sequence of the tags was split in two parts (typically, the first 4 were split from the remaining aminoacids) that were sequentially inserted into the desired sequence using two partially overlapping primer pairs. The list of mutagenesis primers used, PAGE-purified and purchased from Sigma-Aldrich, is reported in supplementary Table S2.

Successful insertion was verified by DNA sequencing of obtained clones. We found the efficiency of tag insertion into proNGF template (6346bp) to be about 33%.

Expression, purification and refolding of proNGF-tag and NGF-tag. wt and tagged proNGF proteins were expressed in *E. coli* and purified using a modified protocol[5] derived from the one previously published for the purification of recombinant human proNGF[7]. We transformed BL21(DE3) *E. coli* with 30-100 ng of plasmid pET11a containing the gene of human proNGF or tagged-proNGF and plated in a Petri dish containing Luria broth (LB) agar supplemented with Ampicillin (Amp) and incubated overnight at 37°C. We then inoculated one colony in 20 ml of LB supplemented with Amp and incubated overnight at 37°C with shaking at 250 rpm. The day after, 18 ml of this culture were inoculated in 1L of LB + Amp at 37°C at 250 rpm until an OD600 of about 1 was reached to obtain a considerable cell mass, before induction with 1 mM addition of isopropyl-b-thio-galactoside (IPTG). The proNGF production was continued at 37°C at 250 rpm shaking. After 5 hours, cell suspension was harvested at 5000 g 4°C for 10 min. The pellet was first resuspended in 20 ml of lysis buffer (10 mM TRIS HCl pH 8, 1 mM EDTA pH 8 and 1mg/ml lysozyme). After sonication (3 cycles of 45” burst followed by 60” pause at 4°C), 50 μg/ml DNase was added. After 30 minutes, the suspension was harvested at 18000 g, 4°C for 30 min and then washed twice with 50 mM TRIS HCl pH 7.5 and 1 mM EDTA. Next, it was harvested again, solubilized with 5 ml of 6M guanidinium, 100 mM TRIS HCl pH 8, 1 mM EDTA and 100 mM DTT, and left overnight dialyzing in a 6M guanidinium pH 3.5 buffer. To allow the correct neurotrophin folding, we measured protein concentration by Bradford assay, followed by a drop by drop addition of 5mg of solubilized inclusion bodies in 100 ml of refolding buffer (1 M Arginine, 100 mM TRIS HCl pH 9,3, 5 mM EDTA pH 8, 1mM Glutathione disulphide (GSSG) and 5 mM Glutathione (GSH)). After an overnight dialysis in phosphate buffer (50 mM Na Phosphate and 1mM EDTA), we purified native and tagged proNGF via an FPLC ion-exchange chromatography. The mature wt

and tagged NGF proteins were obtained upon proteolysis of the respective purified pro-neurotrophin, using trypsin protease (1µg enzyme: 100-200 µg of neurotrophin). In both cases, mature protein was obtained after trypsin cleavage of the propeptide domain up to the N-terminal of mature NGF. Time-course SDS-page experiments were performed to check the correct time needed to obtain mature proteins. A last step of FPLC ion exchange chromatography was used to purify mature neurotrophins from fragments arising from the cleavage of the propeptide domain.

Labeling of tagged proNGF and NGF. To obtain biotin-labelled proNGF and NGF, equal amounts (10 µg) of purified tagged NGF and proNGF were incubated for 30 minutes at 37°C with a reaction mix (10 mM $MgCl_2$, 10 µM CoA-biotin and 2 µM Sfp Synthase (SfpS) or Acp Synthase (AcpS) (New England Biolabs), or no enzyme as control in phosphate buffer up to 30 µl final volume. Untagged proNGF and NGF were subjected to the same reaction as control.

For the fluorolabeling, 10 µg of proNGF-YBBR or NGF-YBBR were incubated for 30 minutes at 37°C at 300 rpm with a reaction mix (10 mM $MgCl_2$, 10 µM CoA-alexa488 or CoA-alexa 647 and 2 µM Sfp Synthase (SfpS) (New England Biolabs), in phosphate buffer up to 250 µl final volume. This step was immediately followed by an ion exchange HPLC chromatography in order to remove the non-fluorescent form from the labeled specie. Chromatographic analyses were performed using a Propac SCX-20 (4x250 mm, Dionex) analytical column (flux 1 ml/min), using these solvents: Sodium Phosphate 100 mM (eluent A)/ Sodium Phosphate 100 mM + NaCl 1M (eluent B), pH 7.4. Purified fluorescent neurotrophins were concentrated using Amicon Ultra-Centrifugal Filter Units 3K and stored up to 10-20 days in pre-coated (BSA 1%) tubes at 4°C.

Determination of labeling yield. In order to determine NGF-YBBR fluorolabeling yield, we compared the integral of the 280 nm peak of fluo-NGF in the chromatogram to the one of the same amount of non-reacted NGF after the correction for the absorbance at 280 nm of Alexa488 fluorophore. Briefly, the correction factor (CF), defined as A_{280nm}/A_{488nm}, was calculated from the HPLC run of Alexa488 fluorophore alone. fluoNGF absorbance at 280 nm was then subtracted of CF multiplied by the fluoNGF absorbance at 488 nm.

PC12, TF1 and DRG culture. PC12 (ATCC, CRL-1721) cells were maintained in a humidified atmosphere at 37°C, 5% CO_2 in RPMI1640 medium supplemented with 10% horse serum, 5% fetal bovine serum and 1% penicillin/streptomycin (Gibco). PC12 differentiation was induced by treatment with 50 ng/ml wt NGF, recombinant tagged NGF, NGF-biotin or NGF-Alexa488 and it was observed after five days.

As a more quantitatively assay for fluo-NGF potency, we used the human erythroleukemic cell line TF1[8]. TF1 cells were cultured in RPMI 1640 medium, 10% fetal bovine serum and 1% penicillin/streptomycin (Gibco), supplemented with 2 ng/ml recombinant human GM-CSF (R&D Systems Inc.). The TF1 proliferation assay was performed in 96 wells microtiter plates by incubating 15,000 cells per well in the presence of several doses of either wild type hNGF and NGF-YBBR or NGF-A4, ranging between 50,000 and 5 pg/ml. Cells were seeded 1h before adding treatments. A MTT Cell proliferation assay (ATCC kit) was employed to evaluate cell response: after a 40-hour culture period, MTT solution was added for additional 4 hours incubation. The intensity of each colorimetric signal was measured at 570 nm in a microtiter plate reader, 16 hours following addition of detergent reagent. In all experiments, each different treatment was done in triplicate.

Rat Dorsal Root Ganlion Neurons (R-EDRG-515 AMP, Lonza) were maintained in a humidified atmosphere at 37°C, 5% CO_2 in Primary Neuron Basal medium (PNBM, Lonza) supplemented with L-glutamine, NSF-1 (2% final concentration) and antibiotics, following the manufacturer's instructions. For the survival of DRG neurons the media was supplemented by 50-100 ng/ml of NGF and it was replaced every 4-5 days with a pre-warmed fresh one, being careful to always leave the main channels filled.

For compartmented cultures, a microfluidic device (Xona Microfluidics, RD 450) was sealed to willCo-wells (WillCo-dish® "Series GWSt-3522) by a plasma bound. The device was coated with a solution of poly-L-Lysine (0.01%, sterile-filtered, P4832, Sigma) and Laminin (5µg/ml, 23017-015,Gibco®, Invitrogen) overnight in a humidified incubator at 37 °C. DRG neurons were plated into the soma chamber (SC). Axons crossed the channel barrier (CC) into the central chamber and reached the axon chamber (AC) within 3–6 days, as previously described[9]. DIV8-15 cultures of DRG neurons were used in all experiments.

Western Blot. In order to quantify proNGF and NGF biotinylation reaction yields, 2 µl of all NGF/proNGF biotinylation reactions were treated under denaturing conditions (100°C, 8 minutes in 2X Laemmli Sample Buffer), loaded on two gels (1 µl for each gel) and electrotransferred to two PVDF membranes respectively.

These were blocked in TBST + 5% w/v BSA, then one of them was blotted with anti-NGF antibody (sc-549, Santa Cruz Biotechnology) (1:2000), while the other one was incubated with HRP-conjugated streptavidin (Zymed®) 1:10000 diluted in blocking solution.

To study the signal transduction effectors, PC12 cells were cultured in P100 petri dish to reach confluence, starved o.n. in a serum-free medium and then incubated with native NGF, tagged NGF, biot-NGF and fluo-NGF (150 ng/ml) at 37°C. After 15 min cells were washed in ice-cold PBS and lysed in RIPA buffer supplemented with proteases and phosphatases inhibitors. 50 µg of each clarified lysate were loaded on a gel and electrotransferred to PVDF membranes. These were first blotted using the antibody anti-Phospho-PLCγ1 (#2821, Cell Signaling Technology;1:1000), anti-Phospho-p44/42 MAPK (#9106, Cell Signaling Technology;1:2000) and anti-Phospho-Akt (#4060, Cell Signaling Technology;1:2000). The primary antibody was detected by using an anti-mouse or rabbit secondary antibody HRP-conjugated (Biorad; 1:1000) diluted in blocking solution. Filters were developed by electrochemiluminescence system.The membranes were then stripped and re-blotted with respectively anti-PLCγ1 (#5690, Cell Signaling Technology;1:2000), anti- p44/42 MAPK (#4695, Cell Signaling Technology;1:1000) and anti-Akt (#4691, Cell Signaling Technology;1:2000). The detection was performed as above described. Presented images have been subjected to linear contrast enhancement after image analysis.

Immunofluorescence (IF). DRG neurons (R-EDRG-515 AMP, Lonza) were grown on microscopy glass coverslips previously coated with Poly-D-lysine and mouse laminin (as described above).

On DIV 10 neurons were put in starvation medium (PNBM complete medium without NGF) for 1 hour before treatment. Medium was then changed to complete medium with 200 ng/ml Alexa647-fluo-proNGF or 108.3 ng/ml Alexa647-fluo-NGF (8.3 nM) and maintained for 1 hr. After treatment, neurons were washed with PBS and fixed for 15' in 4% PFA. Neurons were permeabilzed 7' in 0.1% Triton X-100, 1% BSA. After 5 washes in PBS, neurons were blocked 1h RT in 1%BSA. Rabbit polyclonal anti-TrkA (Millipore 06-574) or anti-p75 (Millipore 07-476) was diluted 1:100 in 4% BSA and put onto neurons for 3 hours at RT on an orbital shaker. After 3 washes in 1%BSA, neurons were incubated 1 hour with 1:100 anti-rabbit secondary antibody conjugated to Alexa 488. Images were acquired with inverted confocal microscope Leica TCS SP5 SMD on DM6000, equipped with MSD module using oil objective HCX PL Fluotar 100X NA 1.30 (Leica Microsystems). Sequential scanning was performed illuminating with a diode laser (Picoquant,

Berlin, Germany) for excitation at 405 nm, with Ar lasers for excitation at 488 nm and 514 nm , and with a HeNe laser for excitation at 633 nm. Images were processed with ImageJ. NT particles were defined to be associated to a receptor if their spots in the two channels were partially or completely overlapped. Statistical analysis was performed with Sigmaplot v.12.0 (SYSTAT).

For double IF, DRG neurons were grown on glass coverslips. DIV 11 neurons were put in starvation for 1hr, then treated 1 hr with 200 ng/ml fluo-proNGF. IF was performed as above, with the following variations: primary antibodies were rabbit polyclonal anti-p75 (Millipore 07-476) 1:100 and mouse anti-TrkA MNAC13[10] to a final concentration of 100μg/ml, whereas secondary antibodies were 1:100 dilutions of anti-rabbit alexa488 and anti-mouse alexa555. Images were processed as above.

Microscopy. All microscopy measurements have been conducted in a wide field microscope (Leica DM6000 equipped with the Leica TIRF-AM module) at 37°C, 5% CO_2. Light has been collected with a HCX PL APO 100x NA 1.47 oil immersion objective (Leica Microsystems) and recorded by an EMCCD camera (Hamamatsu C9100-13). An additional -0.7x lens is mounted in front of the camera chip and a complete field of view of 117 μm x117 μm is obtained. Transmitted light imaging has been performed in differential interference contrast (DIC) configuration. For epi-fluorescence microscopy the solid state lasers at 488nm and 633nm present in the Leica Microsystems TIRF-AM module have been used to excite Alexa488 and Alexa647 respectively. In single-color experiments, Alexa488 fluorescence light has been separated by a dichroic mirror lw_t502lpxr (purchased by Leica) and it has been filtered by a Semrock FF01-525/45 band pass filter (purchased by Leica). The exposure has been set to 0.1 s and a 10 Hz frame rate has been obtained. In two-color experiments, sequential excitations at 488nm and 633 nm are applied and fluorescence light has been separated by the filtercube Quad ET TIRF MC for Laser line 405/488/561/632 No 11523026 (Leica). Light from Alexa488 has been additionally filtered by the Semrock FF01-525/45 band pass filter (in addition to the bandpass capability 525/36 of the Quad filtercube), for the emission for Alexa647 the 705/72 bandpass of the Quad filtercube was sufficient. The exposure time has been set to 0.05 s for each channel and a 6 Hz repetition rate has been obtained. PC12 cells treated with various forms of labeled and tagged NGF, or no NGF as control, were imaged after differentiation at 37°C, 5% CO_2 by the Leica DM6000 microscope capable of transmission DIC imaging.

Single Particle Tracking and Number analysis. Single Particle Tracking analysis has been performed by custom made Matlab scripts following the approach previously described by Raghuveer Parthasarathy[11]. In brief, vesicle sub-pixel localization has been achieved by an analytic, non-iterative calculation of the best-fit radial symmetry center. This localization method has been selected for the following reasons: (i) it provides a very fast localization (up to 180000 frames for each experiment has been analyzed) and (ii) it does not test particle localization on selected spot homogeneity in intensity and/or size, not a proper requirement in our case mainly for the biology of the system under study (possibility of vesicles containing a different number of fluorophores) and to for out-of-focus effects. For each acquired fluorescence image sequence, the channel area has been manually selected, then vesicles localization and trajectories reconstruction has been achieved by means of Matlab code distributed by Raghuveer Parthasarathy. Vesicle trajectories shorter than 10 µm have been discarded in order to minimize the impact of artefacts due to wrong linking. A variable number of trajectories (between ~1000 and ~6000; see Fig. 2 and SI Fig. 4 and 5) have been measured for each selected condition and resulting trajectories have been used to re-center particles for the analysis of the number of fluorophores contained in each spot (number analysis). This analysis is allowed by the 1:1 fluorophore: neurotrophin monomer stoichiometry, and by the identical excitation and emission configuration for all the single fluorophores, which allow to measure the number of fluorophores in each spot by the intensity of the spot. In order to correct for particle movement in the axial direction and for variable background, we quantified the integral of emitted fluorescence by a Gaussian interpolation of "averaged" spots (Fig. 3A): in brief, a 3 µm square selection has been applied in each frame around the particle position and moved frame by frame according to the recorded trajectory. In the image sequence obtained in this way, the recorded vesicle is fixed in the center. Thus a moving average filter of 20 frames could be applied in order to reduce the noise before a Gaussian interpolation is used to quantify the integral of fluorescent light of the selected vesicle. Fitted intensity corresponding to a Gaussian waist more than 600 nm (about twice the optical resolution) has been discarded in order to avoid erroneous brightness estimation due to background and proximity of other vesicles. The obtained intensities are finally normalized by the average value measured with the same algorithm applied to pseudo-immobilized fluorophores contained in a 10% agarose gel. The

average value measured for single fluorophores has been further verified by occasional blinking or photobleaching single steps of fluorophores immobilized in the used gel.

In order to characterize vesicle dynamics, a particle displacement is measured as the displacement from the initial position. For measurements in the axonal compartment the sign of the displacement modulus is positive for retrograde transport and negative for anterograde transport. For the measurement in the SC just positive sign is used. Since the measured trajectories are mostly straight, the average vesicle speed is determined by a fit of displacement versus time (if necessary, along *x* and *y* directions, with the two results quadratically combined); this estimate is more robust against localization uncertainties and linking mistakes at the beginning or the end of a trajectory than the distance from the first to the last position divided by the time delay. However, due to the characteristic stop and go motion exhibited by endosomes, the average vesicle speed doesn't fully describe the movement properties. Thus, in order to better characterize vesicle motion we divided each trajectories in subtrajectories where the vesicle was moving anterogradely, moving retrogradely, or mostly immobile. The separation point is determined by applying a moving average filter of 5 frames in the position of the spots, calculating the instantaneous velocity in each frame, and applying a threshold of 1.25μm/s (-1.25μm/s) for retrograde (anterograde) motion; if a subtrajectory length is 4 frames or less, it is linked with the two surrounding ones (if they are of the same class), or with the closest one with the more similar speed. The threshold was empirically determined by visual inspection of some hundreds of trajectories: the speed on parts that were undoubtedly in a "go" phase was almost always in the 2-3μm/s range (so the threshold was chosen to be half of the mean of this range), with rare cases of "steps" with higher speed (up to 3.7μm/s), and no case of "go" motion with speed below 1.25μm/s. Moreover, the results with the chosen parameters described above were satisfactorily checked on some tens of trajectories. In order to avoid the choice of a somewhat arbitrary threshold, we also measured the distribution of times required to move 1 μm. In brief, particle displacement for each measured trajectory is divided in steps of 1 μm length. For each step, the time delay required to the particle in order to shift 1 μm is measured (the total number of measured time delays corresponds therefore to the total measured displacement in μm). The final distributions are obtained by the histogram of all time delays for all measured trajectories. The mode of the time distribution is refined by interpolating a Gaussian function (with 0 offset) on the 3 (or 4) highest bins, and the recovered value is used to estimate the modal speed during active movement. Moreover, each

measured time delay is used to estimate the speed in the 1 μm related displacement. The obtained speed distribution is shown in Supplementary Fig. 2 in order to visually compare the two different speed distribution. Statistical comparison of speed and time distribution is performed by GraphPad Prism (GraphPad).

Colocalizing trajectories were estimated by visual inspections of extracted trajectories in the green and red fluorescence channels, after an initial automatic selection based on the average time and positions of each trajectory in each microfluidic channel.

Quantification of proNGF intracellular cleavage process. To verify and measure the possibility that fluo-proNGF is cleaved to NGF during the time window of our experiments, PC12 cells were incubated with proNGF, previously fluorolabeled with Alexa 647 (2nM), for different times. As in transport experiments, the precursor NT was administered in fresh medium to the cell culture and maintained at 37° and 5% CO_2. After different incubation times, as reported in Suppl. Fig.7, cells were washed with PBS and lysed in RIPA buffer supplemented with proteases and phosphatases inhibitors. The amounts coming from the lysis of about 400.000 cells treated with fluo-proNGF for different time were loaded on a gel for SDS-PAGE. The gel was imaged using an Image Quant LAS4000 (GE Healthcare) instrument equipped with a narrow bandwidth Led lamp for detection of Alexa 647 fluorescence.

Gene expression experiments: To verify the functionality of both fluo-NGF and fluo-proNGF, we analyzed the gene expression profile induced by NTs in PC12 cells, comparing it with that of wild type neurotrophins. PC12 cells were treated with each neurotrophin's species individually (2nM) for one hour. Then cells were harvested and RNA isolated using TRIzol (Life Technologies) following the standard protocol. RNA was purified using Qiagen RNeasy Mini Kit and the quality assessed by the Bioanalyzer using the 6000 nanoKit (Agilent Technologies). The RNA was then labelled, purified and hybridized using Sure Print G3 Rat Gene Expression v2 8x60K Microarray Kit and One-Color Microarray standard Agilent protocol (version 6.8, June 2015). After washing, chips were scanned using Agilent G2564C scanner with laser resolution of 3 micrometres. Expression data were extracted from TIFF images using Agilent Feature extraction software v 10.7.3.1. Filtering, normalization and further analysis of data have been performed using R-Bioconductor. Data were normalized to the 75th percentile in Log2 scale. Differential mRNAs were selected by the R-

Limma package: differentially expressed mRNAs are those with absolute values of Log2Fold Change ratio> 1.0 and Limma P-value <0.05[12]. Hierarchical clustering of samples was computed by R-hclust package. Principal Component Analisis (PCA) was obtained by the R-prcomp package.

References

1. Cui B, Wu C, Chen L, et al. One at a time, live tracking of NGF axonal transport using quantum dots. *Proc Natl Acad Sci U S A*. 2007;104(34):13666-71. doi:10.1073/pnas.0706192104.

2. Lee R, Kermani P, Teng KK, Hempstead BL. Regulation of cell survival by secreted proneurotrophins. *Science*. 2001;294(5548):1945-8. doi:10.1126/science.1065057.

3. Fahnestock M, Yu G, Coughlin MD. ProNGF: a neurotrophic or an apoptotic molecule? *Prog Brain Res*. 2004;146:101-10. doi:10.1016/S0079-6123(03)46007-X.

4. Pagadala PC, Dvorak L a, Neet KE. Construction of a mutated pro-nerve growth factor resistant to degradation and suitable for biophysical and cellular utilization. *Proc Natl Acad Sci U S A*. 2006;103(47):17939-43. doi:10.1073/pnas.0604139103.

5. Paoletti F, Covaceuszach S, Konarev P V, et al. Intrinsic structural disorder of mouse proNGF. *Proteins*. 2009;75(4):990-1009. doi:10.1002/prot.22311.

6. Marchetti L, De Nadai T, Bonsignore F, et al. Site-Specific Labeling of Neurotrophins and Their Receptors via Short and Versatile Peptide Tags. *PLoS One*. 2014;9(11):e113708. doi:10.1371/journal.pone.0113708.

7. Rattenholl A, Ruoppolo M, Flagiello A, et al. Pro-sequence assisted folding and disulfide bond formation of human nerve growth factor. *J Mol Biol*. 2001;305(3):523-33. doi:10.1006/jmbi.2000.4295.

8. Paoletti F, Malerba F, Bruni Ercole B, Lamba D, Cattaneo A. A comparative analysis of the structural, functional and biological differences between Mouse and Human Nerve Growth Factor. *Biochim Biophys Acta*. 2014;1854(3):187-197. doi:10.1016/j.bbapap.2014.12.005.

9. Park JW, Vahidi B, Taylor AM, Rhee SW, Jeon NL. Microfluidic culture platform for neuroscience research. *Nat Protoc*. 2006;1(4):2128-2136. doi:10.1038/nprot.2006.316.

10. Cattaneo a, Capsoni S, Margotti E, et al. Functional blockade of tyrosine kinase A in the rat basal forebrain by a novel antagonistic anti-receptor monoclonal antibody. *J Neurosci*. 1999;19(22):9687-9697.

11. Parthasarathy R. Rapid, accurate particle tracking by calculation of radial symmetry centers. *Nat Methods*. 2012;9(7):724-6. doi:10.1038/nmeth.2071.

12. Arisi I, D'Onofrio M, Brandi R, et al. proNGF/NGF mixtures induce gene expression changes in PC12 cells that neither singly produces. *BMC Neurosci*. 2014;15(1):48. doi:10.1186/1471-2202-15-48.
